# Supplementary material for: Enabling valley selective exciton scattering in monolayer WSe2 through upconversion
Source: Nat Commun. 2017 Apr 3;8:14927. doi: 10.1038/ncomms14927 (PMC5382264; doi:10.1038/ncomms14927)
Supplement: Supplementary Information — Supplementary Figures, Supplementary Tables, Supplementary Notes and Supplementary References [file ncomms14927-s1.pdf]

Supplementary Note 1 : Experiments

In the main text upconversion and its polarization dependence is studied in detail for ML WSe_2 encapsulated in hexagonal boron nitride (hBN) [1]. This hBN / 1ML WSe_2 / hBN has been chosen for its the spectrally sharp and intense exciton transitions, see Supplementary Fig. 1 bottom panel. But the reported effects are not only observed in encapsulated samples. We also observe very strong upconversion when exciting the A:1s resonantly in standard WSe_2 monolayers directly exfoliated onto SiO_2, see Supplementary Fig. 1 top panel and also in uncovered samples 1ML WSe_2 / hBN (middle panel).

In addition to the A:2s hot PL emission from upconversion we also observe anti-Stokes Raman scattering. This can be seen in Supplementary Fig. 2, where in addition to the A:2s a peak 133 meV above the laser energy is clearly visible in each spectrum, shifting as a function of the laser energy E_L. This is the same data as in Supplementary Fig. 4e in the main text.

In Supplementary Fig. 3 we plot circularly co- and cross-polarized emission in black and red, respectively, of the A:2s upconversion PL. The circular polarization degree P_c is plotted in blue. The polarization of the upconverted emission at the A:2s energy does not originate exclusively from the anti-stokes Raman process, the emission is globally polarized, not just at the Raman energy $E_L + 133$ meV. The A:1s resonance is at 1.723 eV, so the top (bottom) panel shows excitation 3 meV above (2 meV below) resonance. The middle panel corresponds to resonant A:1s excitation. In Fig. 2d of the main text, we measure the emission of the states labelled A:2s and 3s as a function of temperature. In Supplementary Fig. 4 we plot the linewidth, energy separation and intensity ratio of the peaks, extracted from a double peak fit using a Lorentz function.

Supplementary Note 2 : Models

Here we develop a more formal model for the observed optical processes and their polarization selectivity based on the Supplementary Equations (1) - (8). **One-photon absorption.** In linear absorption one absorbed photon generates one exciton. Hence, for resonant excitation of the A:1s state the exciton occupancy $N_{\mathrm{A:1}s}$ is directly proportional to the light intensity I. In the linear regime the exciton generation rate can be conveniently presented as [2]

$$G = \mathcal{A}(1-\mathcal{R})\frac{I}{\hbar\omega}. \tag{1}$$

Here $\mathcal{A}$ is the absorption coefficient of the TMD ML and $\mathcal{R}$ is the reflection coefficient of the

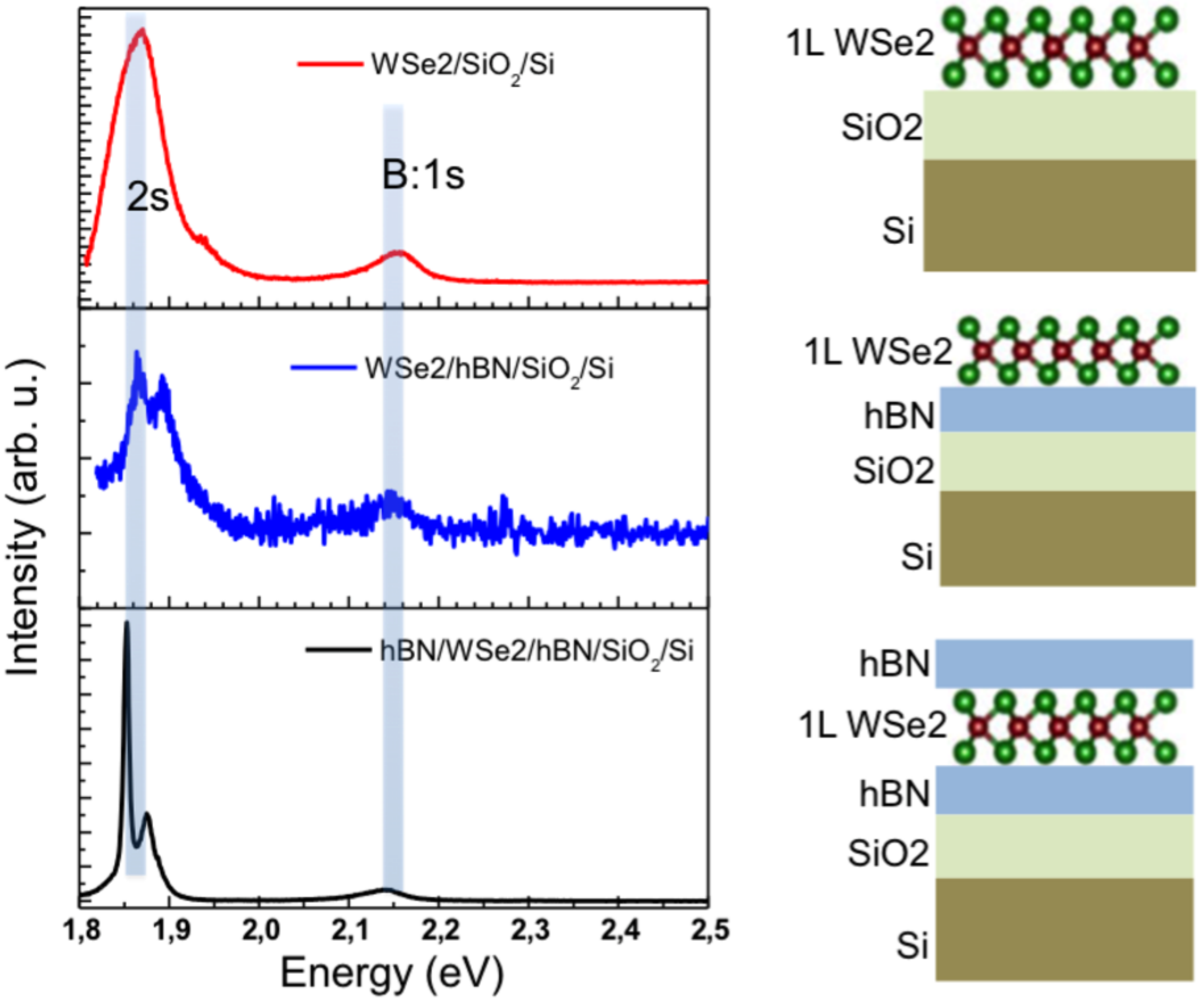

Supplementary Figure 1: In the main text the upconversion and its polarization dependence is studied in detail for hBN / 1ML WSe_2 / hBN (bottom panel) because of the spectrally sharp and intense exciton transitions. But this effects is not linked to encapsulation, very strong upconversion when exciting the A:1s resonantly is also observed for vacuum / 1ML WSe_2 / SiO_2 (top panel), and vacuum / 1ML WSe_2 / hBN (middle panel).

sample, $\hbar\omega$ is the photon energy. Due to nonlinear effects, e.g., absorption saturation, or nonlinear (Auger) recombination of excitons the exciton occupancy can be sublinear function of the incident light intensity. The exciton occupancy

$$N_{\mathrm{A:1}s} \propto G\tau_A \tag{2}$$

where τ_A is the lifetime of the A-exciton. The nonlinearities are either included in the

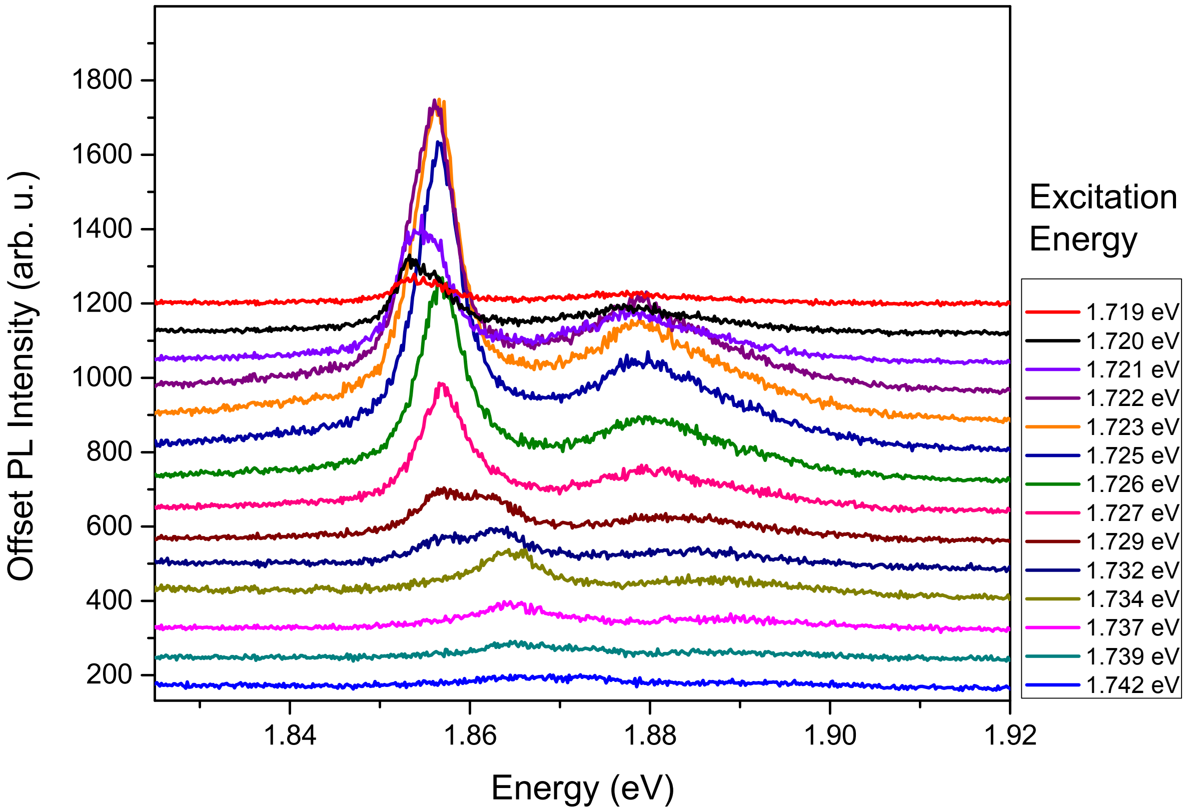

Supplementary Figure 2: Upconversion PL of A:2s state as a function of excitation laser energy. The laser energy E_L is indicated for each spectrum. The data is the same as in Fig.4e in the main text, just plotted in a different format.

intensity dependence of the absorption coefficient $\mathcal{A} = \mathcal{A}(I)$ or in the exciton occupancy dependence of exciton lifetime, $\tau_A = \tau_A(N_{\mathrm{A:1}s})$. The analysis of the particular origin of the sublinear behavior of $N_{\mathrm{A:1}s}$ vs. intensity seen in Fig. 2b of the main text is beyond the scope of the present work.

Two-photon absorption and Auger-like process. The observation of B:1s exciton emission at A:1s excitation is possible if any only if, in addition to the photogenerated exciton, another quantum: second exciton, photon or phonon is involved. Otherwise the energy conservation law is violated. Let us consider in more detail the processes without phonon involvement.

Standard two-photon absorption (2PA) involves a *virtual* intermediate state. In this case the generation rate of the excitons in the highly excited states denoted as $|f\rangle$ in Fig. 2c

of the main text (energy equals to $2\hbar\omega$, i.e. twice the laser energy) is proportional to the square of incident radiation intensity [3, 4, 5]

$$G_f^{\mathrm{2PA}} \propto I^2. \tag{3}$$

In the studied situation the single photon energy $\hbar\omega$ equals to the A:1s-exciton energy. Hence, the intermediate state for the two-photon process can be real. The two-photon excitation via real state (RS-2PA) can be viewed as a two-step process: creation of the A-exciton at the first step and transition of the A-exciton to the excited $|f\rangle$ state via the second photon absorption. On the level of free electron-hole pairs the process is illustrated in Supplementary Fig. 5. In this process the generation rate of the excited excitons takes the form

$$G_f^{\mathrm{RS-2PA}} \propto N_{\mathrm{A:1}s} I. \tag{4}$$

This dependence is weaker than I^2 due to possible saturation of linear absorption. Particularly, if intermediate states saturate $G_f^{\mathrm{RS-2PA}} \propto I$.

Supplementary Table I: Comparison of upconversion intensities for various phonon-less mechanisms: 2PA is the two-photon absorption via virtual state, RS-2PA is the two-photon absorption involving real intermediate states, Supplementary Fig. 5, "sat" denotes saturation of the real intermediate state, Auger is the Auger-like two-exciton process, Supplementary Fig. 6.

Process	2PA	RS-2PA	RS-2PA (sat)	Auger
Intermediate state	virtual	real	real<br>intermediate states saturate	
Generation rate	$\propto I^2$	$\propto N_{\mathrm{A:1}s} I$	$\propto N_{\mathrm{A:1}s}^{(\mathrm{sat})} I (\propto I)$	$\propto N_{\mathrm{A:1}s}^2$

Moreover, two photons can be absorbed independently resulting in formation of two A:1s-excitons as schematically shown in Supplementary Fig. 6a. The Coulomb interaction between the charge carriers forming the excitons results in the redistribution of the excitation in the $\boldsymbol{k}$- and energy-spaces. Particularly, the Auger-like process is possible. In this scenario due to the Coulomb scattering one of the interacting electrons goes to the unoccupied state in the valence band, while another one takes the released energy and gets promoted to the excited energy band [6]. As a result, the excited $|f\rangle$ state of the electron-hole pair is formed, Supplementary Fig. 6b. The strong Coulomb interaction between the electron and

the hole results in the spread of excitonic functions in the $\boldsymbol{k}$-space and relaxes the momentum conservation law. For the Auger-like process the generation rate of excitons in the excited states is quadratic in the occupancy of A:1s excitons

$$G_f^{\text{Auger}} \propto N_{\text{A:1}s}^2. \tag{5}$$

Supplementary table I summarizes the results of the analysis performed above on the dependence of the exciton generation rate on the incident laser intensity. We assume that the relaxation from $|f\rangle$ exciton states towards A:2s- and B:1s-excitons is linear,i.e., its rate is proportional to the first power of the $|f\rangle$ states occupancy. Therefore, the upconversion intensity is proportional to the generation rate of the excited excitons, G_f. The comparison of the experimentally observed upconversion PL intensity as a function of the laser power, Fig. 2b of the main text, with the suggested mechanisms shows that the Auger-like process is the plausible source of the upconversion. The 2PA via real states is also possible if the substantial saturation of the intermediate states is assumed. However, the rise of the temperature up to the room temperature keeps upconversion efficient, Fig. 2d of the main text. This rules out trap states, which usually play a role of intermediate states for RS-2PA in the quantum well structures [7], as the origin of the intermediate states here.

Bose stimulation in the relaxation process. In order to illustrate the build-up of the cross-circular polarization we present the rate equation model accounting for the valley-independent generation of B-excitons via relaxation from excited $|f\rangle$ states and valley-dependent stimulated scattering towards A:1s excitons. To that end we introduce the generation rate of B-excitons, which is the same in both σ^+ and σ^- polarizations, G_{B} and present the rate equations for the densities $N_{\text{B}}^{\pm}$ of the $\sigma^{\pm}$-polarized B:1s-excitons in the form

$$\frac{dN_{\text{B}}^+}{dt} + \frac{N_{\text{B}}^+}{\tau_B} + W(1+N_{\text{A}}^+)N_{\text{B}}^+ = G_B, \tag{6a}$$

$$\frac{dN_{\text{B}}^-}{dt} + \frac{N_{\text{B}}^-}{\tau_B} + W(1+N_{\text{A}}^-)N_{\text{B}}^- = G_B, \tag{6b}$$

where $N_{\text{A}}^{\pm}$ are the occupancies of $\sigma^{\pm}$ polarized A:1s-excitons, τ_B is the lifetime of B-excitons irrelated with relaxation towards the A-states, W describes the rate of the relaxation to A:1s-excitonic state. This description is simplified as we neglect spin/valley relaxation of excitons and, moreover, the exciton relaxation from B- to A-state can be with multiple steps, in which case a cascaded process may be relevant [8]. The circular polarization degree of

B-excitons can be expressed, in the limit $W\tau_B(1+N_{\rm A}^{\pm}) \ll 1$ as

$$P_{\rm B} = \frac{N_{\rm B}^{+} - N_{\rm B}^{-}}{N_{\rm B}^{+} + N_{\rm B}^{-}} = -\frac{1}{2} W\tau_B N_{\rm A:1s} P_{\rm A}, \tag{7}$$

where $P_{\rm A} = (N_{\rm A}^{+} - N_{\rm A}^{-})/N_{\rm A:1s}$ is the circular polarization of the A-excitons, $N_{\rm A:1s} = N_{\rm A}^{+} + N_{\rm A}^{+}$ is the total number of A-excitons. Clearly, the polarization P_B of the upconverted B:1s-exciton emission is reversed as compared with $P_{\rm A}$ and it is the larger the more excitons are created by the laser, i.e. the larger $N_{\rm A:1s}$. For the incident intensity $I = 100$ μW/μm^2 and A-exciton lifetime $\tau_A = 1$ ps we have, in accordance with Eq. (1) the A-exciton density $N_x = 10^{10}$ cm^{-2}. To obtain an estimate of the occupancy of a single quantum state we present

$$N_{\rm A:1s} = \frac{\hbar^2 N_x}{M\Delta}, \tag{8}$$

where $M \approx 0.5 m_0$ with m_0 being free electron mass, is the exciton effective mass and Δ is the energy width of exciton distribution. The right hand side of Eq. (8) plays a role of degeneracy parameter. For fully thermalized excitons $\Delta = k_B T \approx 0.3$ meV, $N_{\rm A:1s} \approx 0.05$. However, the thermalization does not take place under resonant excitation conditions and low temperatures because the exciton-acoustic phonon scattering is relatively weak in TMD materials and strongly exceed the radiative lifetime of excitons [9]. The lowest limit for Δ comes from the laser linewidth, which is extremely narrow in our experiments with *cw* excitation. Taking $\Delta = 3 \times 10^{-2}$ meV and $W\tau_B = 0.5$ (evaluation of these quantities is beyond the scope of the present paper) and $P_A = 100$ % we have for $P_B = -15\%$ in the order of magnitude agreement with experiment.

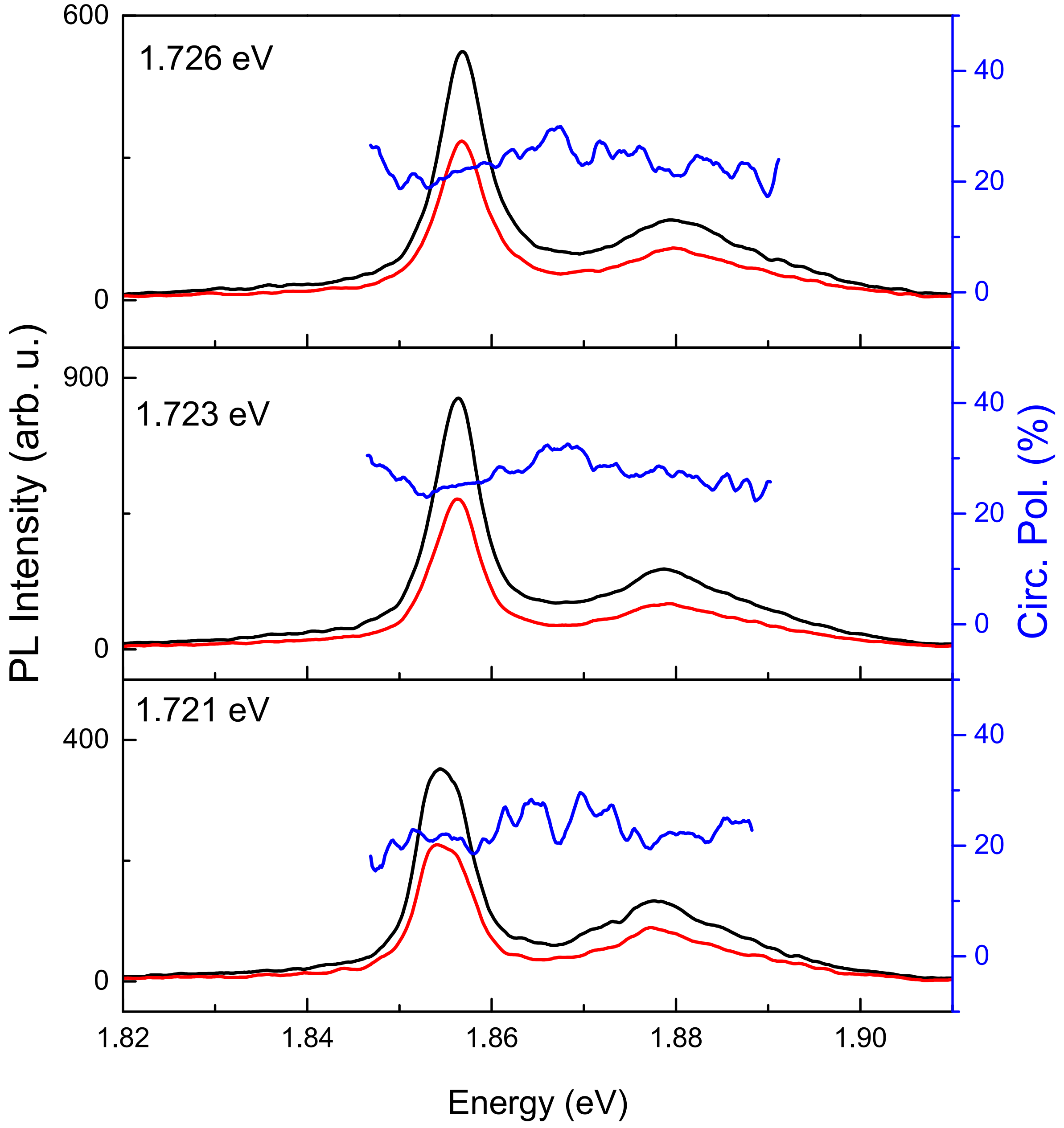

Supplementary Figure 3: Circularly polarized excitation, co-circular detection (black) and cross-circular (red) detection of A:2s upconversion PL at T=4K signal (left axis). The circular polarization degree is plotted in blue (right axis). The laser energy E_L is indicated for each panel.

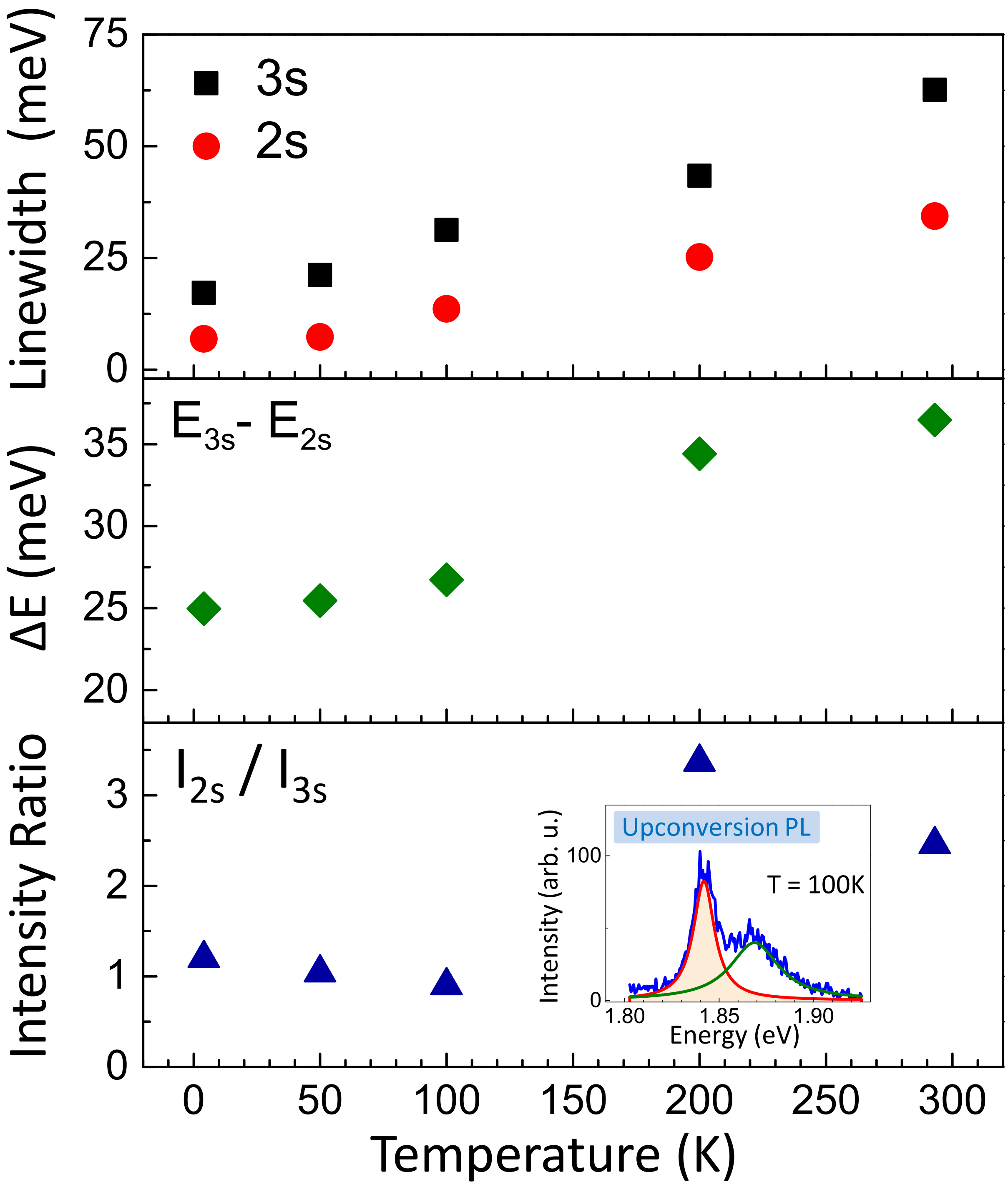

Supplementary Figure 4: Same date as Fig. 2d in the main text. Linewidth (top panel), energy separation between $2s$ and $3s$ (middle panel), intensity ration $2s$: $3s$ emission (bottom panel). The inset shows a typical upconversion PL spectrum.

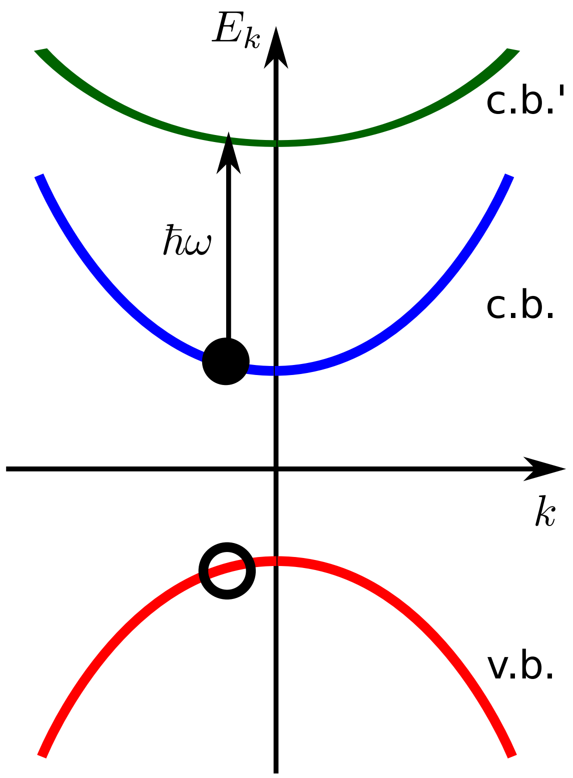

Supplementary Figure 5: Sketch of the two-photon excitation with a real intermediate state. Open circle denotes the empty state in the valence band (v.b.), filled circle denotes occupied state in the conduction band (c.b.). The arrow denotes the transition to the final state (in the remote conduction band c.b.') through absorption of a second photon. The Coulomb interaction induces spread of the electron-hole pair in $\boldsymbol{k}$-space (not shown) relaxing the momentum conservation law. The band gaps and dispersion are not shown to scale.

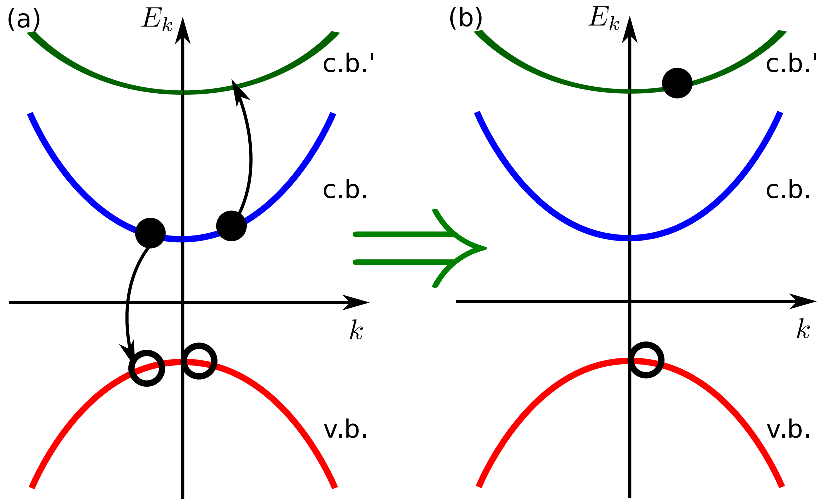

Supplementary Figure 6: Sketch of the Auger-like two-exciton process. (a) Initial "two A-exciton state" formed by independent absorption of two photons. Arrows denote the transitions induced by the Coulomb interaction between electrons. (b) Final state of the electron-hole pair with electron occupying excited band (c.b.'). Other notations are the same as in Supplementary Fig. 5

Supplementary References

[1] Taniguchi, T. & Watanabe, K. Synthesis of high-purity boron nitride single crystals under high pressure by using ba-bn solvent. *Journal of Crystal Growth* **303**, 525 – 529 (2007).

[2] Seidel, W., Titkov, A., André, J. P., Voisin, P. & Voos, M. High-efficiency energy up-conversion by an "auger fountain" at an inp-aiinas type-ii heterojunction. *Phys. Rev. Lett.* **73**, 2356–2359 (1994).

[3] Ivchenko, E. L. *Optical spectroscopy of semiconductor nanostructures* (Alpha Science, Harrow UK, 2005).

[4] Berkelbach, T. C., Hybertsen, M. S. & Reichman, D. R. Bright and dark singlet excitons via linear and two-photon spectroscopy in monolayer transition-metal dichalcogenides. *Phys. Rev. B* **92**, 085413 (2015).

[5] Glazov, M. M. *et al.* Intrinsic exciton-state mixing and nonlinear optical properties in transition metal dichalcogenide monolayers. *Phys. Rev. B* **95**, 035311 (2017).

[6] Abakumov, V. N., Perel, V. I. & Yassievich, I. N. *Nonradiative recombination in semiconductors* (North Holland, Amsterdam, 1991).

[7] Hellmann, R. *et al.* Low-temperature anti-stokes luminescence mediated by disorder in semiconductor quantum-well structures. *Phys. Rev. B* **51**, 18053–18056 (1995).

[8] Liew, T. C. H. *et al.* Proposal for a bosonic cascade laser. *Phys. Rev. Lett.* **110**, 047402 (2013).

[9] Slobodeniuk, A. O. & Basko, D. M. Exciton-phonon relaxation bottleneck and radiative decay of thermal exciton reservoir in two-dimensional materials. *Phys. Rev. B* **94**, 205423 (2016).
